# Tetralogy of Fallot and pheochromocytoma in a situs inversus totalis: An unusual association

**DOI:** 10.15171/jcvtr.2016.27

**Published:** 2016-09-30

**Authors:** Rubén Kevin Arnold Tapia-Orihuela, Jorge Huaringa-Marcelo, David Loja-Oropeza

**Affiliations:** ^1^Universidad Nacional Mayor de San Marcos, Facultad de Medicina de San Fernando, Lima, Perú; ^2^Sociedad Científica de San Fernando, Lima, Perú; ^3^Hospital Nacional Arzobispo Loayza, Lima, Perú; ^4^Universidad Nacional Federico Villarreal, Facultad de Medicina, Lima, Perú

**Keywords:** Tetralogy of Fallot, Pheochromocytoma, Congenital Heart Defects, Situs Inversus, Hypoxia

## Abstract

**Introduction:** Situs inversus totalis is an uncommon anomaly which exist a complete transposition of organs and it’s occasionally associated with congenital heart diseases, such as tetralogy of fallot. Pheochromocytoma is a rare neuroendocrine tumor with an annual incidence of 2-8 cases per million people and for years has been studied its relationship with the hypoxic pathway.

**Case Report:** A 29 year old male with a history of tetralogy of fallot corrected at 10 years and situs inversus totalis. He was admitted to hospital with a progressive story of four months of constipation, palpitations, headache, dyspnea and sweating. Physical examination revealed a thinned man with peripheral cyanosis, clubbing and signs of decompensated congestive heart failure as hepatomegaly, legs edema, multifocal systodiastolic murmurs, abdominal distension and jugular venous distention. The echocardiogram shows severe right ventricular dysfunction and severe pulmonary hypertension. Furthermore, abdominal computed tomography shows right adrenal mass. Elevated metanephrines and catecholamines confirmed the diagnosis of pheochromocytoma. Surgical removal is decided and preoperative management begins with alpha-adrenergic blockade, however the patient had a hemodynamic decompensation with an unfavorable evolution.

**Discussion:** In conclusion, there are few reports of cyanotic congenital heart disease with pheochromocytoma. Several studies show a significant association between both of them due to chronic hypoxia leads sustained hyperresponsiveness in adrenal medulla and it would cause the tumor. Special preoperative management of pheochromocytoma is recommended when there underlying heart disease and congestive heart failure. We present the first international report of tetralogy of fallot and pheochromocytoma in a patient with situs inversus totalis.

## Introduction

Situs inversus totalis is an unusual finding characterized by the complete transposition of thoracic and abdominal organs with an incidence in the general population between 1:10 000 to 1:20 000 adults.^1^ This condition is typically associated with a normal life expectancy unless a gastrointestinal or cardiac anomaly is present.^2^ A 2%-5% incidence of congenital *heart* disease (CHD) is observed in situs inversus totalis^1^ and tetralogy of Fallot (TOF) is the most common cyanotic congenital heart disease (CCHD)^3^.

Pheochromocytomas are rare catecholamine-secreting neuroendocrine tumors arising from adrenomedullary chromaffin cells and they occur in 2-8 cases per million people per year.^5^ These tumors can be sporadic or inherited, and a quarter of sporadic cases have a known genetic mutation.^6^ They are often diagnosed accidentally in asymptomatic patients and about 5% to 6.5% of incidentalomas are pheochromocytomas^7^.

There are few reported cases of neuroendocrine tumors such as pheochromocytoma with CCHD in Japan, USA and India.^8-10^ The relationship between both of them has been speculated in the past^8,11^; however, a recent study has shown a strong link between CCHD and pheochromocytoma, this association can be due to chronic hypoxic stress, genetic factors or some combination of both.^12^ We present the first international report of TOF and pheochromocytoma in a patient with situs inversus totalis.

## Case Report

A 29-year-old Peruvian patient presented to the hospital with progressive history of constipation, bloating and oppressive abdominal pain in the left upper quadrant for 4 months. Then, 1 month ago middle effort dyspnea, palpitations, orthopnoea and sweating were added. The last 15 days appear headache and bilateral lower limb edema, further exacerbation of previous symptoms, so hospitalization is decided.

He had a history of palliative operation at 4 months and 8 years of age, and a curative operation at 10 years of age. Then after one year he began arrhythmias therefore a permanent pacemaker was implanted on his abdomen, this pacemaker was changed at the age of 21 because of battery depletion. Also, he was diagnosed of situs inversus totalis at 3 months old. No history of situs inversus or CHD and other diseases in the family. He was born preterm at 28 weeks and presented a low psychomotor development due to underlying heart disease.

**Figure 1:**
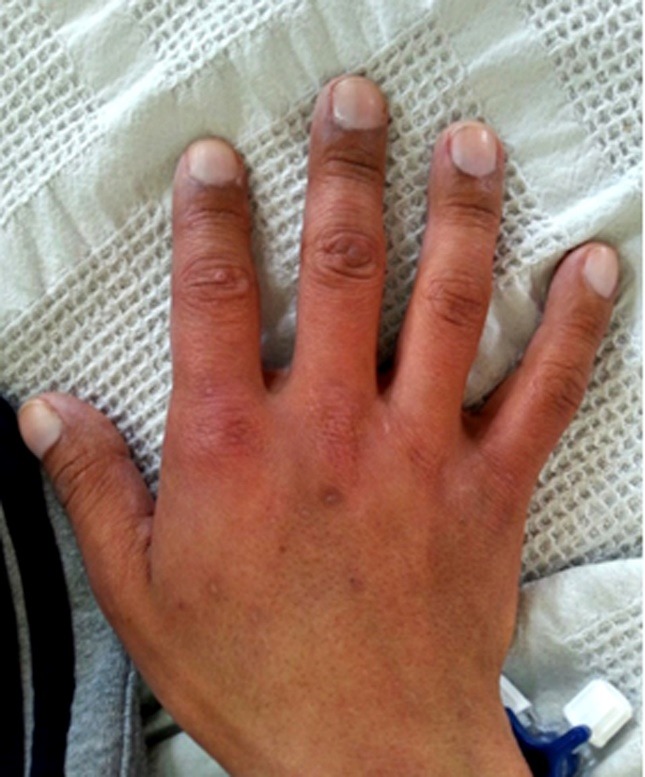
Peripheral cyanosis and clubbing due to persistent chronic hypoxia.

**Figure 2:**
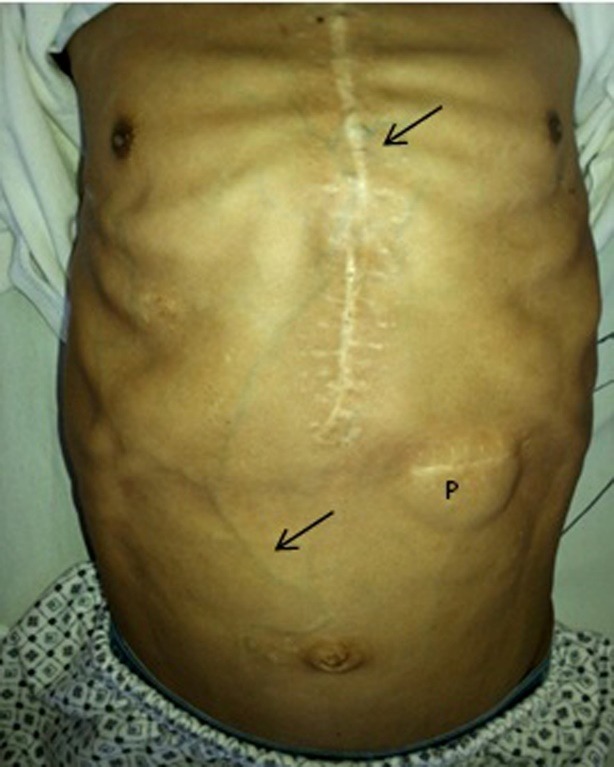
Thoracoabdominal collateral circulation (black arrows). Furthermore, abdominal distention, old surgical scars and implanted pacemaker (P).

Physical examination showed a patient with short stature. On admission, he was breathing at 20 breaths per minute with a blood pressure of 110/70, pulse of 76 and oxygen saturation of 92%. Also peripheral cyanosis, clubbing (Figure 1), leg swelling ++/++++, mild pallor, moderate macroglossia, non-palpable thyroid, thoracolumbar scoliosis, bilateral jugular engorgement, holosystolic murmur in focus aortic IV/VI, systolic-diastolic murmur in pulmonary focus IV/VI and systolic murmur in mitral focus IV/VI. In addition, patient had abdominal distention, abdominal pain on light and deep palpation in the left upper quadrant, thoracoabdominal collateral circulation and hepatomegaly 5cm below the left costal margin (Figure 2).

Transthoracic echocardiography showed TOF repaired, severe pulmonary hypertension, severe dilation of the right side, left ventricular dysfunction with LVEF 45%, and severe right ventricular dysfunction with severe tricuspid regurgitation. Abdominal ultrasound showed gallstones and multiple hepatic nodules suggestive of nodular chronic liver disease.

Results of thyroid hormones showed free* T4* 0.76 pg/mL (range: 0.8-1.8 pg/mL), free T3 1.5 pg/mL (range: 2-4.5 pg/mL) and TSH ultrasensitive 5.39 uUI/mL (range: 0.5-5 uUI/mL), leading to the diagnosis of primary hypothyroidism. Treatment was initiated with levothyroxine 12.5 mg/d oral, having an adequate clinical response decreasing constipation. Other relevant laboratory findings showed Hb 9.2 g/dL (range: 13.5-17 g/dL), cortisol 24 ug/dL (range: 6.2-26 ug/dL) discarding the presence of an adrenal carcinoma, *gamma*-*glutamyl transferase* 119 U/L (range: 11-55 U/L), Na 135 mEq/L (range:135-145 mEq/L), K 4.4 mEq/L (range:3,5 a 5,3 mEq/L) and parathormone 35 pg/mL (range: 15-65 pg/mL).

**Figure 3:**
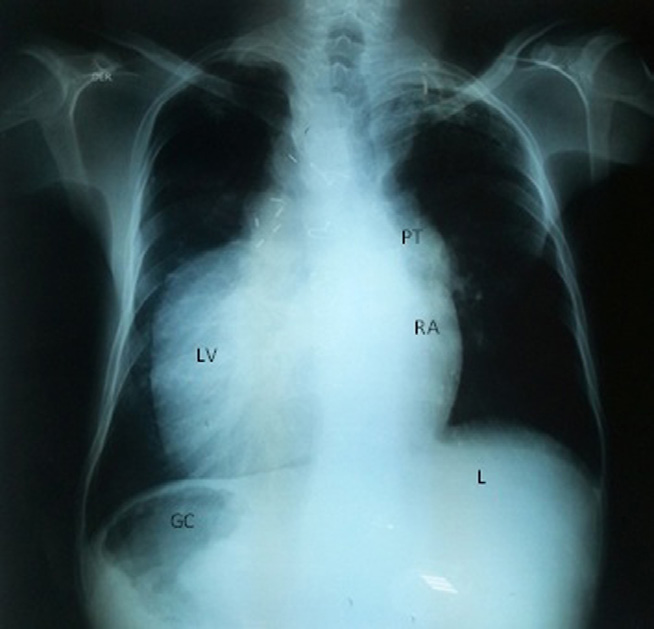
Chest radiograph reveals dextrocardia, cardiomegaly, an enlarged pulmonary trunk (PT), scoliosis and situs inversus. Gastric chamber (GC) on the right side and liver (L) on the left side.

**Figure 4:**
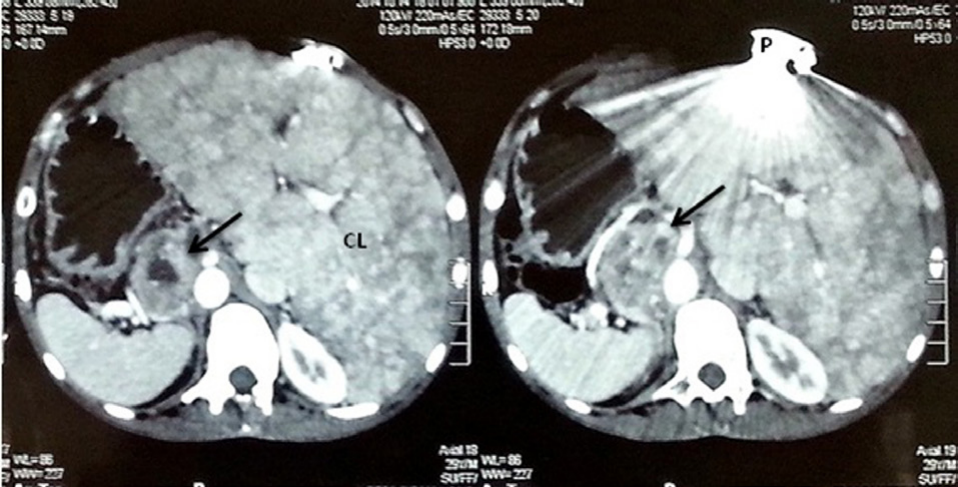
Abdominal computed tomography shows situs inversus and right adrenal mass of 52 mm x 33 mm x42 mm (black arrows). Congestive liver (CL) shows changes consistent with cirrhosis secondary to severe right heart failure. P (Pacemaker).

Chest radiograph reveals dextrocardia, cardiomegaly and an enlarged pulmonary trunk (Figure 3). Abdominal computed tomography concludes chronic diffuse liver disease with multiple hepatic nodules, however a mass (52 mm × 33 mm × 42 mm) was found incidentally in right adrenal gland (Figure 4). Also, metanephrines in urine and plasma epinephrines showed elevated levels (Table 1), which confirmed the diagnosis of pheochromocytoma.

**Table 1:** Urinary and plasmatic levels of metanephrines and epinephrines

	** Results**		** Normal range**
Urine metanefrine (mg/24h)	37.28		0.05 – 1.00
Plasma fractionated catecholamines (pg/mL	Norepinephrine	690.2	150-750
	Epinephrine	77.3	10-60
	Total catecholamines	780.8	<860

Surgical resection of pheochromocytoma was decided. Ten days before surgery, alpha-adrenergic blocking was started with terazosin 2.5 mg/d oral, however it was suspended the eighth day of therapy because of the patient presented hemodynamic decompensation and hypoxemic respiratory failure. Also, the procalcitonin levels were elevated, chest x-ray showed cotton-like infiltrates and finally doctors conclude septic shock due to nosocomial pneumonia. The evolution was unfavorable and the patient died in the next three days.

## Discussion

Situs inversus totalis is a rare condition that causes thoracoabdominal organs to be positioned in a mirror image. It is mostly asymptomatic, autosomal recessive inheritance^13^ pattern and is associated in a small percentage (2% to 5 %) to the development of CHD as TOF,^3^ representing the leading causes of morbidity and mortality in all of the anomalies associated with situs inversus.^14^

Patients with TOF develop progressive hypoxemia in the first years of life and without palliative or corrective surgery do not usually survive to adulthood.^15^ Furthermore, if the time of correction extends, they might have sequels that predispose to arrhythmias, right ventricular dilation and dysfunction, leading to a tricuspid regurgitation.^16^ In late-stage, signs and symptoms of chronic congestive heart failure appear, which showed the patient on admission and during hospitalization was cataloged as congestive heart failure NYHA class III. Likewise, should be considered the triggering factors of heart failure as infections, arrhythmias, pulmonary embolism, hyperthyroidism, hypertensive crisis, neuroendocrine tumors, among others.^17^

Pheochromocytomas are tumors arising from chromaffin cells that develop in adrenal medulla and can be sporadic or inherited, about 25% of sporadic cases have a genetic mutation.^18^ 10% of these tumors are associated with neurofibromatosis type I, multiple endocrine neoplasia type 2 (MEN2), von Hippel-Lindau disease and familial paraganglioma syndrome.^19^ In our patient, the presence of cutaneous neurofibromas, hyperparathyroidism, medullary thyroid carcinoma, Lisch nodules, neuromas mucocutaneous, neurological abnormalities and family history was discarded; therefore, he was not classified in any of these syndromes.

Pheochromocytoma is a rare tumor with a highly variable presentation; even symptoms may be absent or unrecognized. Cardinal symptoms include headache, palpitations, sweating and hypertension.^18^ It should be considered that some signs and symptoms of pheochromocytoma can be masked by underlying heart disease or congestive heart failure, presented by the patient. Also, pheochromocytoma was diagnosed incidentally by abdominal CT and later confirmed by urinary and plasma high levels of catecholamines and their metabolites.

Some reports of pheochromocytoma in patients with CCHD have been reported for decades,^8^ predominantly cases from the United States India and Japan.^8-10^ This is the first case worldwide where converge TOF and pheochromocytoma in a patient with situs inversus totalis.

Neural crest cells are related to the formation of the outflow tracts of the adrenal medulla and the heart.^20^ Up to a dozen inherited genetic mutations have been identified in pheochromocytoma-associated syndromes listed above, which include mutations in the *VHL*, *RET*, *NF1*, *SDHx*, and *SDHAF2* genes. Most of these have in common a cellular pathway that activates hypoxia inducible factors as hypoxia-induced factor, which promotes angiogenesis, glycolysis and cell proliferation.^21,22^ The hypoxic state stimulates catecholamine secretion from the adrenal medulla, and sustained hyperactivity may lead to hyperplasia and neoplasia of this endocrine gland. Several studies have suggested the possibility of tumor induction such as pheochromocytoma due to the hypoxic condition in CCHD.^11,12,28^

HIF-1α and HIF-2α target genes are overexpressed in some genes of pheochromocytoma, suggesting that exists an HIF deregulation in this tumor^23^ and moreover, the blood level of HIF1α is high in patients with CCHD.^24^

Our patient has 10 years of sustained hypoxia because of late surgical correction of TOF, and despite it being corrected; the sequels continued to some degree this hypoxia, for example developing right heart failure and severe pulmonary hypertension.^9^ Because of that, patient persisted with hypoxemia and he also presented clubbing, a chronic hypoxia sign. In a case series, the 87% of patients with cardiac anomalies associated with neuroendocrine tumors are those leading to cyanosis.^11^ On the other hand, abdominal venous engorgement found in our patient, was mainly as secondary to cor pulmonale and portal hypertension as a result of post-hepatic cardiac cirrhosis.

Also, it is possible to find patients with history of severe cyanosis and complete surgical correction of TOF without neuroendocrine tumors. This may be due to low hypoxemia by appropriate repair timing, genetic factors or possibly they never were diagnosed because symptoms and signs were masked specially by the intense cyanosis and vasodilatation of TOF. Thereby, the complete mechanism of CHD leading to pheochromocytoma development has yet to be revealed.

De la Monte et al^11^ reported that 4 of 15 patients with CHD and neuroendocrine tumors, had TOF. Three of which developed pheochromocytomas and they found a statistically significant correlation between CHD and neuroendocrine tumors. As well a recent study^25^ evidence that 5 of 17 patients with CCHD and pheochromocytoma had TOF; and the mean age at the time of diagnosis of pheochromocytoma was 24.1 years, younger than the mean age (40 years) of patients with pheochromocytoma without other diseases. This dissimilarity propose that systemic chronic hypoxia stimulates the development of pheochromocytoma in younger patients.

A current multicenter cases series^12^ whose objective was to test the hypothesis that chronic hypoxia in patients with CCHD increases the risk of pheochromocytoma and paraganglioma shows a strong association between these rare entities. In accordance with this study, patients with CCHD have a greater risk of developing pheochromocytoma or paraganglioma (OR: 6.0) than patients with non-CCHD (OR: 0.9). Likewise, other studies suggest an increased risk of developing parangangliomas and pheochromocytomas in people living at a high altitude.^26,27^

We recommend taking special care in the preoperative management of pheochromocytoma in patients with congestive heart failure and underlying heart disease. The chronic congestive heart failure had a fundamental role in the worsening of patient because of it leaded a hemodynamic decompensation in the pre-surgery days. Note that currently there are no established guidelines for preoperative management of pheochromocytoma normotensor or with comorbidities.^19,28^ Furthermore, neuroendocrine tumors should be considered as possibility of decompensation of heart failure, paying crucial attention when there is an underlying disease as TOF.

In conclusion, congenital cyanotic heart diseases such as TOF, may be associated with other anomalies such as Situs inversus totalis, and in the context of diseases with chronic hypoxia (such as cyanotic CHD), patients could develop neuroendocrine tumors as a pheochromocytoma.

## Ethical approval

The authors have obtained all permission before using any data and patient images.

## Competing interests

All authors declare no competing financial interests exist.
